# Field component modulation of vortex beams in uniaxial crystals driven by angular and topological charge dependencies

**DOI:** 10.1038/s41598-025-32473-1

**Published:** 2026-01-24

**Authors:** Aldsoky Albadry, Mamdouh Shams El-Din, Mohamed Nawareg

**Affiliations:** Department of Physics, Faculty of Science, Damietta University, Damietta, 34511 Egypt

**Keywords:** Applied optics, Optical physics, Nonlinear optics

## Abstract

We investigate the propagation of linearly polarized vortex–Gaussian beams carrying topological charge *M* through a rutile uniaxial crystal at arbitrary angles relative to the optical axis. Using a full vectorial numerical model, we provide a systematic mapping of how both the propagation angle $\theta$ and the topological charge jointly govern the evolution of the transverse and longitudinal electric–field components. The results reveal a pronounced and angle-dependent modulation of all field components, accompanied by a strong and predictable amplification with increasing *M*. In particular, the longitudinal component exhibits an *M*-dependent oscillatory behavior that peaks near orthogonal incidence, while the generated transverse component reaches its maximum close to parallel propagation. The phase distributions show a clear topological imprint, including a reduction of the longitudinal-field phase winding to $(M-1)$ due to anisotropy-driven coupling. These observations shed light on the coupled roles of anisotropy, propagation angle, and vortex charge in shaping the vectorial structure of light inside uniaxial crystals. The results hold relevance for applications in optical manipulation, vector-beam generation, and quantum and classical information processing.

## Introduction

Vortex beams (VBs), a fascinating class of structured light, are distinguished by their helical phase fronts described by $e^{- iM \phi }$, where *M* represents the topological charge–an integer quantifying the phase singularity’s strength and $\phi$ is the azimuthal coordinate^1^. This unique phase structure leads to *M* intertwined helical phase fronts, culminating in a central phase singularity, which manifests as a dark core in the beam’s intensity profile^1,2^. Beyond their spatial characteristics, VBs carry orbital angular momentum (OAM), a property directly linked to the topological charge. The azimuthal phase dependence induces a circulating Poynting vector, resulting in an OAM of $M \hbar$ per photon along the propagation axis. From a quantum mechanical perspective, the photon’s state can be represented as a superposition of $2 M + 1$ OAM eigenstates, ranging from $- M \hbar$ to $M \hbar$, highlighting the multifaceted nature of OAM in these beams^3,4^.

The mathematical framework for describing VBs often relies on Laguerre-Gaussian (LG) modes, denoted as ${LG}^{M}_p$, which form a complete and orthogonal basis in Hilbert space^4,5^. This comprehensive description allows for the generation and manipulation of high-dimensional quantum states, leveraging the integer-valued topological charge. Notably, Allen et al. demonstrated that the OAM carried by VBs is independent of polarization, distinguishing it from spin angular momentum, which is intrinsically linked to circular polarization^2,3^. While the fundamental origin of OAM in light can be traced to multipole transitions in atomic and molecular systems, the practical generation of VBs has become routine in modern optical laboratories, enabling a wide array of applications^1,6–9^.

The interaction of VBs with anisotropic media, particularly uniaxial crystals, opens up a rich landscape for light manipulation. These crystals, characterized by refractive indices that vary with polarization and propagation direction, offer enhanced control over vortex beam properties compared to isotropic materials^10,11^. The anisotropic nature of uniaxial crystals facilitates polarization conversion, a crucial process for generating vector vortex beams (VVBs) with tailored polarization states^12^. In addition, these crystals can mitigate self-focusing in high-power laser systems, making them valuable for applications like STED microscopy and optical trapping, where precise control over beam intensity and polarization is paramount^12^. Moreover, the pulsed laser interaction with anisotropic media provides a new perspective to laser writing in crystalline materials^9^. The ability to manipulate OAM states within uniaxial crystals is also pivotal for advancing quantum information technologies, enabling the development of sophisticated quantum communication and processing systems^12^.

The propagation of VBs through uniaxial crystals has been extensively studied, with significant emphasis on configurations where the beam travels either orthogonal or parallel to the crystal’s optical axis^7–9,13–22^. These investigations have revealed a rich variety of phenomena, including the emergence of longitudinal field components, modifications to amplitude and phase structures, polarization evolution, and the generation or transformation of optical vortices. For example, Ref.^7^ demonstrates both theoretically and experimentally the mechanisms of polarization and topological-charge conversion for Gaussian beams propagating parallel to the optical axis, while Ref.^8^ provides analytical and experimental validation of complex vector-beam formation under on-axis propagation. Furthermore, Ref.^9^ examines beam evolution at oblique angles, highlighting the strong sensitivity of polarization and intensity distributions to the inclination relative to the optical axis.

Despite these advances, the combined influence of propagation angle and topological charge on the full vectorial field structure–particularly the interplay between transverse and longitudinal components–remains insufficiently explored^23–26^. This gap is especially relevant given the strong anisotropy of uniaxial media and the pronounced sensitivity of vortex beams to angular deviations. A detailed and systematic investigation over a continuous range of $(M,\theta )$ is therefore needed to fully resolve the vectorial dynamics and to elucidate how anisotropy-driven coupling mechanisms shape the evolution of structured light in such media.

In contrast to previous analyses that typically focused on either on-axis propagation or fixed angular configurations, the present work provides a unified and systematic mapping of how both the propagation angle $\theta$ and the topological charge *M* jointly determine the evolution of all electric-field components ($E_x$, $E_y$, $E_z$). We identify and quantify the reduction of the longitudinal-field phase winding to $M-1$, trace this behavior to gradient-induced coupling mechanisms, and reveal a topological-charge–dependent amplification of the longitudinal component that strongly interacts with the propagation angle. To the best of our knowledge, these combined vectorial features across the $(M,\theta )$ parameter space have not been previously reported.

The results presented here have direct implications for crystal-based vector-beam generation, spin–orbit and orbital–orbital coupling processes, structured-light engineering in anisotropic media, and optical manipulation schemes requiring precise control over field components.

In the following, we first outline the theoretical framework and derivations, then present and discuss the main results together with their physical interpretation. Finally, we briefly comment on the experimental feasibility and possible verification of the predicted effects.

## Theory

### Linearly polarized paraxial beams propagating at an arbitrary angle through uniaxial crystals

The propagation characteristics of a monochromatic paraxial beam traversing a uniaxial crystal can be determined by solving the vectorial wave equation under the paraxial approximation. A theoretical model developed in^27^ investigated the arbitrary propagation of such beams by calculating the intensities of the transverse field components. Furthermore, the evolution of the beam’s polarization and spin angular momentum was analyzed in^11^. The spectral description of an arbitrarily propagating beam was provided by a model presented in^28^.

**Fig. 1:**
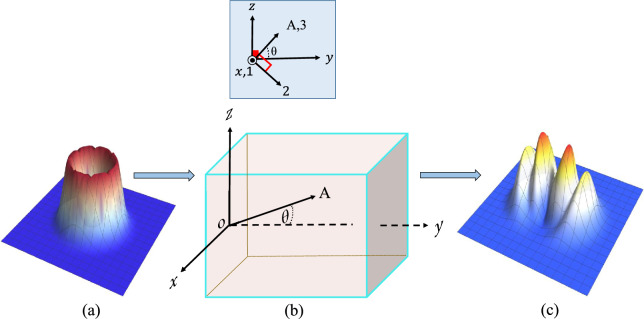
(**a**) Intensity of the incident linearly polarized vortex-Gaussian beam with topological charge *M*. (**b**) Uniaxial-crystal geometry showing the optical axis (OA), propagation direction *y*, and transverse axis *x*. The principal axes are: 1-axis along *x*, 3-axis along the OA, and 2-axis perpendicular to the *x*–OA plane. The angle $\theta$ denotes the beam’s inclination relative to the OA. (**c**) Intensity profile of the output beam after propagation through the crystal.

Uniaxial crystals are significant optical media with diverse applications, including the fabrication of wave retarders and optical compensators. They also serve as sources for generating entangled quantum states. A uniaxial crystal is characterized by its optical axis (*OA*). The principal coordinate system of the crystal is defined by three orthogonal axes, one of which aligns with the optical axis (*OA*). In this work, these axes are denoted as *OA*, the *x*-axis, and the third axis perpendicular to the *xOA* plane (see Fig. 1). In this principal coordinate system, the dielectric tensor $\mathbf {\epsilon }$ of the uniaxial crystal is given by^11,27^:

1 $$\begin{aligned} \mathbf {\epsilon }= \begin{pmatrix} n_o^2 & 0 & 0 \\ 0 & n_o^2 & 0 \\ 0 & 0 & n_e^2 \end{pmatrix} \end{aligned}$$

where $n_o$ and $n_e$ represent the ordinary and extraordinary refractive indices, respectively, with $n_e$ being the refractive index along the optical axis (*OA*).

This study investigates the arbitrary propagation of a light beam along the *y*-direction through a uniaxial crystal. The beam is incident normally on the crystal’s *xz*-plane (input surface) and its propagation direction makes an angle $\theta$ with the crystal’s optical axis. The primary goal is to determine the resulting electric field components ($E_x$, $E_y$, $E_z$) in the laboratory coordinate system. This specific configuration, involving both arbitrary propagation and normal incidence, is of particular interest due to its diverse applications, notably in the generation of quantum entangled states at non-zero propagation angles. Furthermore, understanding the beam’s behavior under these linear propagation conditions is a fundamental prerequisite for exploring more complex nonlinear optical phenomena such as down-conversion and wave mixing within uniaxial crystals.

Based on the uniaxial dielectric tensor in Eq. (1), solving the vectorial wave equation yields three electric field components ($E_1$, $E_2$, $E_3$) corresponding to the crystal’s principal coordinate system in which $\mathbf {\epsilon }$ is defined. Here, $E_3$ is the field component along the optical axis (*OA*), $E_2$ along the axis perpendicular to the *x*–OA plane and $E_1$ in the *x*-direction. The relationship between the field components in the principal coordinate system ($E_1$, $E_2$, $E_3$) and the laboratory coordinate system ($E_x$, $E_y$, $E_z$) is given by^11^:

2 $$\begin{aligned} E_{x} ({\textbf{r}}_{\bot }, y)&= E_1 ({\textbf{r}}_{\bot }, y), \nonumber \\ E_{y} ({\textbf{r}}_{\bot }, y)&= E_2 ({\textbf{r}}_{\bot }, y) \sin \theta + E_3 ({\textbf{r}}_{\bot }, y) \cos \theta , \nonumber \\ E_{z} ({\textbf{r}}_{\bot }, y)&= E_3 ({\textbf{r}}_{\bot }, y) \sin \theta - E_2 ({\textbf{r}}_{\bot }, y) \cos \theta , \end{aligned}$$

where ${\textbf{r}}_{\bot } = (x, z)$ represents the transverse coordinates. These electric field components ($E_x$, $E_y$, $E_z$) are dependent on the propagation angle $\theta$. Consequently, the components in the (*x*, *z*, *y*) coordinate system directly correspond to the components in the crystal’s principal coordinate system in two specific cases:

1. For $\theta = 0^\circ$ (propagation along the optical axis): $E_{y} \rightarrow E_3$ and $E_{z} \rightarrow - E_2$.
2. For $\theta = 90^\circ$ (propagation perpendicular to the optical axis): $E_{y} \rightarrow E_2$ and $E_{z} \rightarrow E_3$.

Although ($E_1$, $E_2$, $E_3$) are defined in the crystal’s principal coordinate system, they can be expressed in any coordinate system, such as (*x*, *y*, *z*), as shown in Eq. (2). As demonstrated in^11^, $E_1$ and $E_2$ contain contributions from both ordinary and extraordinary waves within the crystal, whereas $E_3$ depends solely on the extraordinary wave. Consequently, the components $E_x$, $E_y$, and $E_z$ are influenced by both ordinary and extraordinary fields, as will be evident in the subsequent equations. The total electric field can be expressed as:

3 $$\begin{aligned} {\textbf{E}} ({\textbf{r}}_{\bot }, y) = {\textbf{E}}_x ({\textbf{r}}_{\bot }, y) + {\textbf{E}}_{y} ({\textbf{r}}_{\bot }, y) + {\textbf{E}}_{z} ({\textbf{r}}_{\bot }, y). \end{aligned}$$

Based on the solutions for ($E_1$, $E_2$, $E_3$) presented in^11,27^, together with the transformations in Eq. (2), the electric field components in the laboratory frame can be written in terms of inverse Fourier transforms as follows:

4 $$\begin{aligned} E_{x} ({\textbf{r}}_{\bot }, y)&= \frac{1}{4\pi ^{2}} {\mathscr {F}}^{- 1} \left[ \frac{ \beta _o \beta _e \; \text {e}^{\eta _o} + k_x^2 \; \text {e}^{\eta _e}}{k_x^2 +\beta _o \beta _e } \, E ({\textbf{k}}_{\bot }) \right] , \nonumber \\ E_{y} ({\textbf{r}}_{\bot }, y)&= \frac{1}{4\pi ^{2}} {\mathscr {F}}^{- 1} \left[ \frac{ - k_x \beta _e \sin \theta \; \text {e}^{\eta _o} + k_x (\delta \cos \theta + \beta _e \sin \theta ) \; \text {e}^{\eta _e}}{k_x^2 +\beta _o \beta _e } \, E ({\textbf{k}}_{\bot }) \right] , \nonumber \\ E_{z} ({\textbf{r}}_{\bot }, y)&= \frac{1}{4\pi ^{2}} {\mathscr {F}}^{- 1} \left[ \frac{ k_x \beta _e \cos \theta \; \text {e}^{\eta _o} + k_x (\delta \sin \theta - \beta _e \cos \theta ) \; \text {e}^{\eta _e}}{k_x^2 +\beta _o \beta _e } \, E ({\textbf{k}}_{\bot }) \right] , \end{aligned}$$

Here, $E({\textbf{k}}_{\bot })$ denotes the Fourier transform of the input field ${\textbf{E}}(x,0,z)$, where ${\textbf{k}}_{\bot }=(k_x,k_z)$ represents the transverse wave-vector components, and ${\mathscr {F}}^{-1}$ is the inverse Fourier transform operator. This formulation provides a direct and compact description of the components ($E_x$, $E_y$, $E_z$) for any normally incident beam on the *xz*-plane that is linearly polarized along the *x*-axis–perpendicular to the crystal’s principal plane, which contains the optical axis and the propagation direction *y*.

The exponential factors appearing in Eq. (4) are defined by

5 $$\begin{aligned} \eta _o&= i (n_o k_0 - \beta )\, y, \nonumber \\ \eta _e&= i \alpha (1 - b)\, y. \end{aligned}$$

To facilitate the interpretation of these expressions, the additional parameters are given by

6 $$\begin{aligned} b&= \frac{k_x^2 + k_{z}^2 \left[ 1 + \left( \frac{n_{e }^2}{n_{o }^2} - 1 \right) \sin ^2 \theta \right] }{2 n_e^2 k_0^2 }, \end{aligned}$$

and the quantities $\beta _o$ and $\beta _e$ that couple the ordinary and extraordinary components are

7 $$\begin{aligned} \beta _o&= k_{yo}\sin \theta - k_z\cos \theta , \nonumber \\ \beta _e&= k_{ye}\sin \theta - k_z\cos \theta . \end{aligned}$$

The ordinary-dispersion term $\beta$ and the extraordinary scaling parameter $\alpha$ are expressed as

8 $$\begin{aligned} \beta&= \frac{k_x^2 + k_z^2}{2 n_o k_0}, \nonumber \\ \alpha&= \frac{n_e k_0}{\sqrt{\frac{n_e^2}{n_o^2}\cos ^2\theta + \sin ^2\theta }}, \end{aligned}$$

while the parameter $\delta$, arising from the extraordinary-wave decomposition, is given by

9 $$\begin{aligned} \delta&= \frac{(k_x^2 + k_{z}^2 + k_{ye}^2) n_o^2 k_0^2}{\left( 1 - \frac{n_e^2}{n_o^2}\right) (k_{ye}\cos \theta + k_z\sin \theta )}. \end{aligned}$$

Under the paraxial approximation, the ordinary and extraordinary longitudinal wave-vector components follow^27^:

10 $$\begin{aligned} k_{yo}&= n_o k_0 - \beta , \nonumber \\ k_{ye}&= \frac{n_e k_0}{\sqrt{\left( \frac{n_e^2}{n_o^2} - 1 \right) \cos ^2\theta + 1}} (1 - b). \end{aligned}$$

This framework shows that the propagation characteristics of a linearly *x*-polarized beam–i.e., a beam whose polarization is perpendicular to the principal plane–can be rigorously analyzed for arbitrary propagation angles relative to the optical axis of a uniaxial crystal. Solving Eqs. (4) therefore enables the determination of the beam evolution at any output plane $y=L$ (the crystal thickness) in a medium characterized by refractive indices $n_o$ and $n_e$.

### Vortex Gaussian beam propagation

Consider an incident vortex Gaussian beam located in the *xz*-plane and linearly polarized along the *x*-direction (see Fig. 1). This beam represents the fundamental mode of Laguerre-Gaussian beams and can be mathematically described as^29^:

11 $$\begin{aligned} {\textbf{E}} (x, 0, z) = (x + i z)^M \, \text {e}^{- \frac{k_0(x^2 + z^2)}{2 Z_r}} \hat{e}_x . \end{aligned}$$

where *M* is the topological charge of the vortex beam, $Z_r$ is the Rayleigh length, and $k_0$ is the wave number of the incident beam.

The first term of Eq. (11) can be expressed in a summation form as^30^:

12 $$\begin{aligned} (x + i z)^M = \sum _{l = 0}^M \frac{{z}^l (i^l M!) x^{M - l}}{l! (M - l) !}, \end{aligned}$$

where *l* is an integer ranging from 0 to *M*.

Specifically, for the vortex-Gaussian beam defined in Eq. (11), the Fourier transform $E ({\textbf{k}}_{\bot })$ can be solved analytically for the given parameters. However, the integral expressions in Eq. (4) are generally analytically intractable. Consequently, a computational program was developed to numerically evaluate these equations.

To illustrate the propagation characteristics and to compare the crystal’s influence on Gaussian ($M = 0$) and vortex Gaussian ($M \ne 0$) beams, the relative intensities of the total transverse, and longitudinal components, as well as their phases, are defined as:

13 $$\begin{aligned} S_0 (x,y,z)&= |E_x(x,y,z)|^2 + |E_{z}(x,y,z)|^2, \nonumber \\ S^r_0 (x,y,z)&= \frac{|E_x(x,y,z)|^2 + |E_{z}(x,y,z)|^2}{\max [S_0 (x,0,z)] }, \nonumber \\ S_i (x,y,z)&= |E_{i}(x,y,z)|^2 \qquad \qquad \qquad \qquad ; i = z, y, \nonumber \\ S^r_i (x,y,z)&= \frac{ |E_{i}(x,y,z)|^2}{\max [S_0 (x,y,z)] } \qquad \qquad \qquad ; i = z, y, \nonumber \\ \phi _i (x,y,z)&= \arg [E_{i}(x,y,z)] \qquad \qquad \qquad ; i = x, y, z, \end{aligned}$$

Here, $S^r_0$ represents the transverse intensity of the propagating beam with topological charge *M*, normalized to the maximum intensity of the input vortex beam with the same charge. Furthermore, at an output face at ($y=L$), $S^r_{z}$ and $S^r_{y}$ denote the intensities of the transverse and longitudinal components, respectively, normalized to the maximum intensity of the total transverse component $S_0(x,y,z)$. This normalization allows for the assessment of their relative contributions compared to the overall transverse intensity.

## Results and discussion

This study numerically investigates the propagation of Vortex Gaussian Beams (VGBs) through a 1000 $\mu$m thick slice of rutile, a uniaxial crystal. VGBs are characterized by a helical wavefront and carry orbital angular momentum, quantified by an integer known as the topological charge, denoted by *M*. We solved the propagation equations (Eq. 4) to simulate the beam’s evolution as it travels through the crystal at various angles ($\theta$) relative to the crystal’s optic axis. We compare the behavior of VGBs with different topological charges ($M = 1, 2, 3, 4$) against the standard Gaussian beam ($M = 0$). The primary goal is to understand how the anisotropic crystal modifies the beam’s intensity profile and phase structure, particularly focusing on the influence of the topological charge *M* and the propagation angle $\theta$. To quantify these changes, we analyze the following relative intensity components: the transverse intensity profile of the output beam ($S^r_0$), the intensity of the electric field component along the *z*-axis ($S^r_z$), transverse to propagation and the intensity of the electric field component along the *y*-axis ($S^r_y$), longitudinal (i.e., the propagation direction). The *z*- and *y*-components arise due to the interaction of the initially *x*-polarized beam with the anisotropic crystal structure.

**Fig. 2:**
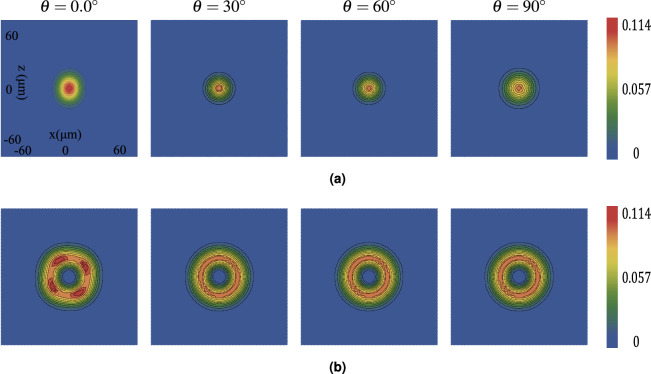
Relative transverse intensity ($S_{0}^r$) distribution of beams with topological charges (**a**) $M = 0$ and (**b**) $M = 2$ propagating through a uniaxial rutile crystal (thickness = $1000~\mu \text {m}$, $n_o = 2.5837$, $n_e = 2.8719$) at different propagation angles $\theta = 0^\circ$, $30^\circ$, $60^\circ$, and $90^\circ$. The incident beam parameters are $\lambda = 0.6328~\mu \text {m}$ and the beam’s radius equals $5~\mu \text {m}.$

Figure 2 displays the calculated transverse intensity profiles ($S^r_0$) for a standard Gaussian beam ($M=0$, panel a) and a VGB with $M=2$ (panel b) after propagating through the crystal at different angles ($\theta$). The beam profile becomes elliptical after passing through the crystal. This distortion is most significant when the propagation angle $\theta$ is $0^{\circ }$. As $\theta$ increases towards $90^{\circ }$, the beam shape becomes less elliptical and more circular. The maximum value of the relative transverse intensity ($S^r_0$) is slightly higher at $\theta = 0^\circ$ compared to other angles. For the $M=0$ beam, the peak $S^r_0$ is 0.1136 at $\theta = 0^\circ$, while for the $M=2$ beam, it is 0.107.

**Fig. 3:**
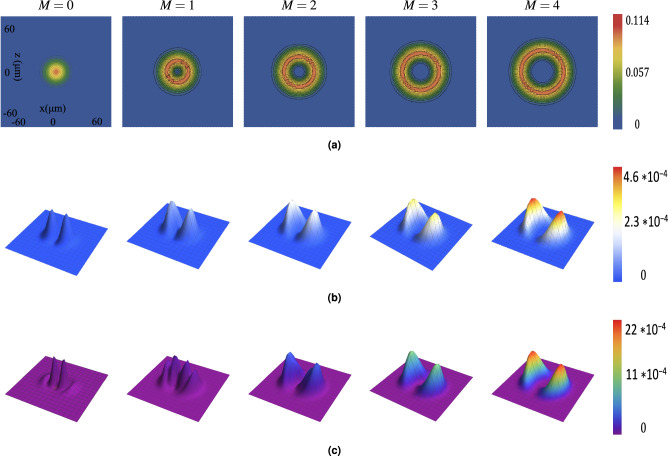
Effect of topological charge *M* on the intensity distributions at $\theta = 30^\circ$ for: (**a**) transverse component $S^{r}_{0}$, (**b**) *y*-component $S^{r}_{y}$, and (**c**) *z*-component $S^{r}_{z}$. Results are shown for $M = 0$, 1, 2, 3 and 4. Crystal and beam parameters are identical to those in Fig. 2.

Table 1 compares the input beam radius ($R_M$) with the output radius after propagation through the crystal (at $\theta = 30^\circ$). The radius $R_M$ is defined as the distance from the beam center (for $M=0$) or the ring center (for $M > 0$) to the point where the intensity drops to $1/e^2$ (approximately 13.5%) of its maximum value. For vortex beams ($M \ne 0$), this definition yields two radii; we consistently consider the outer radius. Propagation through the 1000 $\mu$m crystal causes significant beam expansion for all topological charges. While the absolute size increases with *M*, both at input and output, the ratio of the output radius to the input radius is nearly constant across different values of *M*, approximately 3.34. This scaling is also visually apparent in Fig. 3(a).

**Table 1:** Beam radius ($R_M$) in $\mu \text {m}$ for different topological charges *M*, measured at the point where intensity equals $1/e^2$ of the maximum value. The input beam radius represents measurements in free space, while the output beam radius shows measurements after propagation through a $1000~\mu \text {m}$ thick rutile crystal at $\theta = 30^\circ$. For vortex beams ($M \ne 0$), the outer radius is reported.

Beam charge *M*	Input beam radius ($\mu$m)	Output beam radius ($\mu$m)
0	5	17.166
1	7.504	25.42
2	8.868	29.836
3	9.942	33.269
4	10.85	36.294

**Fig. 4:**
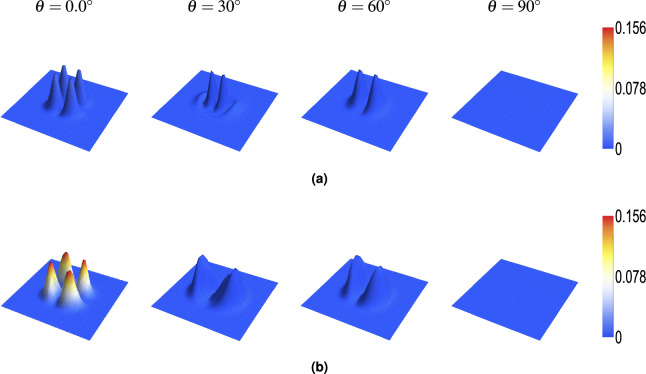
*z*-component intensity ($S_{z}^r$) distribution for beams with topological charges (**a**) $M = 0$ and (**b**) $M = 2$ propagating through a uniaxial rutile crystal at various propagation angles $\theta$. Crystal parameters: thickness = $1000~\mu$m, $n_o = 2.5837$, $n_e = 2.8719$. Beam parameters: input beam waist $s_0 = 5~\mu$m, wavelength $\lambda = 0.6328~\mu$m.

**Fig. 5:**
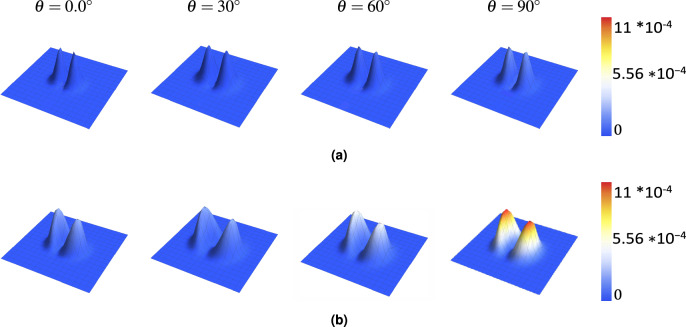
*y*-component intensity ($S_{y}^r$) distribution of beams with topological charges (**a**) $M = 0$ and (**b**) $M = 2$ propagating through a uniaxial rutile crystal at different propagation angles $\theta$. Crystal and beam parameters are identical to those in Fig. 2.

The interaction with the anisotropic crystal generates electric field components along the *y*- (longitudinal) and *z*- (transverse) directions, whose relative intensities are $S^r_y$ and $S^r_z$. Figures 4 ($S^r_z$) and 5 ($S^r_y$) show the spatial distribution of these components for $M=0$ and $M=2$ at various angles $\theta$. The peak intensity of these components varies significantly with $\theta$. As shown for $S^r_z$ (Fig. 4, Table 2), the peak intensity exhibits a damped oscillatory behavior as $\theta$ changes from $0^{\circ }$ to $90^{\circ }$, being maximal near $\theta = 0^\circ$ and minimal (zero) at $\theta = 90^\circ$. Conversely, $S^r_y$ has its smallest value at $\theta = 0^\circ$ and largest at $\theta = 90^\circ$ (Fig. 5). Increasing the topological charge *M* significantly enhances the intensity of both the $S^r_z$ and $S^r_y$ components. For instance, comparing $M=0$ and $M=2$ at $\theta = 0^\circ$ (from Fig. 4), the maximum $S^r_z$ increases dramatically from $\sim$0.01 to $\sim$0.15. Similarly, at $\theta = 90^\circ$ (from Fig. 5), $S^r_y$ increases from $\sim$0.00017 for $M=0$ to $\sim$0.0011 for $M=2$, becoming substantially larger for higher *M* (e.g., reaching $\sim$0.00046 for $M=4$ at $\theta =30^\circ$ as shown in Fig. 3b, also up to $\sim$0.002 at $\theta =90^\circ$ ). The spatial structure of these components also depends on *M*. For example, $S^r_z$ (Fig. 3c) exhibits ring structures. For $M=0$, two semi-rings are prominent. As *M* increases, the outer ring intensifies while the inner ring diminishes, nearly disappearing by $M=4$.

**Table 2:** Maximum values of the relative *z*-component intensity ($S^r_{z} \times 10^{-4}$) at different propagation angles $\theta$ for $M = 0$, demonstrating the damped oscillatory behavior of the component with respect to propagation angle.

$\theta$	$0^\circ$	$15^\circ$	$30^\circ$	$45^\circ$	$60^\circ$	$70^\circ$	$90^\circ$
Max $S^r_z (\times 10^{-4})$	104.65	22.06	0.477	1.736	3.02	2.88	0

**Fig. 6:**
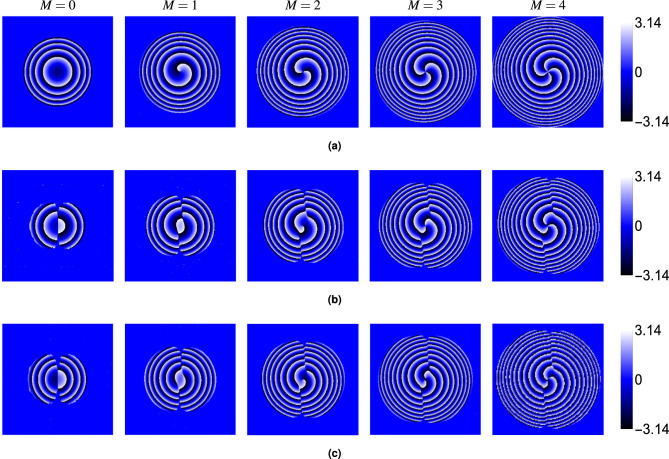
Phase distributions of (**a**) $E_x$, (**b**) $E_y$, and (**c**) $E_z$ components for beams with topological charges $M = 0$, 1, 2, 3 and 4 propagating at $\theta = 30^\circ$ through the rutile crystal.

**Fig. 7:**
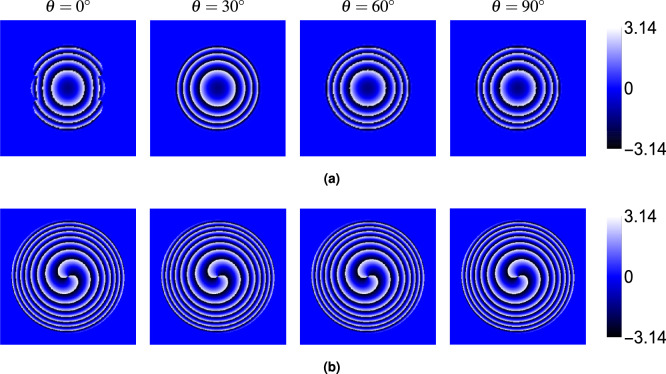
Phase distributions of the $E_x$ component for beams with topological charges (**a**) $M = 0$ and (**b**) $M = 2$ at different propagation angles $\theta = 0^\circ$, $30^\circ$, $60^\circ$, and $90^\circ$. Crystal and beam parameters are identical to those in Fig. 2.

**Fig. 8:**
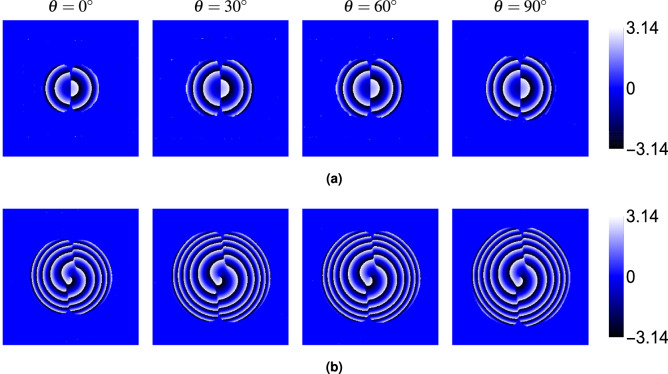
Phase distributions of the $E_y$ component for beams with topological charges (**a**) $M = 0$ and (**b**) $M = 2$ at different propagation angles $\theta = 0^\circ$, $30^\circ$, $60^\circ$, and $90^\circ$. Crystal and beam parameters are identical to those in Fig. 2.

**Fig. 9:**
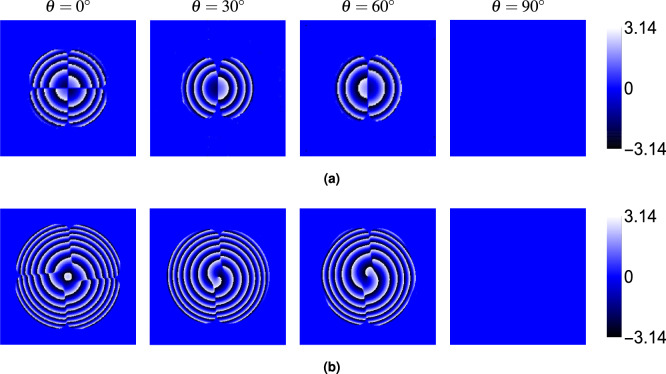
Phase distributions of the $E_z$ component for beams with topological charges (**a**) $M = 0$ and (**b**) $M = 2$ at different propagation angles $\theta = 0^\circ$, $30^\circ$, $60^\circ$, and $90^\circ$. Crystal and beam parameters are identical to those in Fig. 2.

Finally, we examined the phase ($\phi$) of the electric field components ($E_x$, $E_y$, $E_z$). Figure 6 (at $\theta = 30^\circ$) shows that the phase structure carries a distinct signature of the topological charge. The phase of the dominant *x*-component ($\phi _x$, panel a) displays *M* intertwined helical ramps, characteristic of an *M*-charge vortex beam. The phases of the crystal-induced components ($\phi _y$, $\phi _z$, panels b and c) exhibit $M-1$ intertwined helical ramps centered in the beam (for $M \ge 1$). For $M=0$, the phase is relatively uniform or shows simple curvature. Figures 7, 8 and 9 show the phases $\phi _x$, $\phi _y$ and $\phi _z$, respectively, for $M=0$ and $M=2$ at different angles $\theta$. While the intensity of the $E_y$ component varies strongly with $\theta$ (as seen in Fig. 5), the fundamental structure of its phase (number of spirals) remains largely unaffected by the propagation angle $\theta$. The same holds true for $\phi _x$ and $\phi _z$.

Collectively, the propagation of Vortex-Gaussian beams through a uniaxial crystal produces several distinctive effects: the beam undergoes angular-dependent ellipticity, exhibits substantial transverse expansion, and generates additional longitudinal ($E_y$) and transverse ($E_z$) field components through anisotropy-driven vectorial coupling. The strengths of these induced components are highly sensitive to both the propagation angle $\theta$ and the topological charge *M*, with higher charges significantly enhancing their amplitudes. Despite these intensity and structural modifications, the characteristic spiral phase associated with the input vortex is preserved in the dominant $E_x$ component, while the induced components acquire an $(M-1)$ helical phase structure arising from gradient-mediated coupling inside the crystal as will be discussed in the next subsection.

Importantly, these results provide a coherent picture of how angular deviation and topological charge jointly govern the evolution of all field components–a regime that has not been systematically explored in earlier studies focused on either on-axis or fixed-angle propagation. The combined trends identified here, including the charge-dependent amplification of the longitudinal field and the robustness of the $(M-1)$ phase structure across the full angular range, offer practical insight for structured-light control in birefringent media. This joint $(M,\theta )$ mapping establishes a useful framework for designing crystal-based vector-beam generators, tailoring spin–orbit and orbital–orbital coupling processes, and guiding future experimental investigations of vortex-beam propagation under arbitrary inclination.

### Physical origin of angular and topological modulation

The modulation of the transverse and longitudinal field components arises from the anisotropic vectorial coupling imposed by the uniaxial crystal. The longitudinal component $E_y$ reaches its maximum near $\theta = 90^\circ$, where the propagation direction becomes nearly orthogonal to the optical axis (OA). In this configuration, the incident *x*-polarized ordinary field acquires a strong projection onto the extraordinary refractive-index surface whose refractive index depends on the angle between the wave vector and the OA. This enhanced projection increases birefringent cross-polarization coupling and strengthens the extraordinary-wave walk-off, producing a pronounced longitudinal field through vectorial mixing.

Conversely, the transverse crystal-induced component $E_z$ exhibits its maximum near $\theta = 0^\circ$, when the beam propagates close to the OA. Under these conditions, Maxwell’s transversality condition, $\nabla \!\cdot \!{\textbf{E}} = 0$ forces strong coupling from the vortex-dependent transverse gradients into the transverse $E_z$ component. Since this coupling is mediated by spatial derivatives of the dominant $E_x$ field, its magnitude grows with increasing topological charge *M*.

The core physical interpretation regarding the $M \rightarrow M-1$ reduction relies on two fundamental principles: the divergence-free nature of the electric field (Maxwell’s equation) and the algebraic action of the spatial derivative (the gradient operator) on the helical phase front. The key equations describing the phenomena, can be given as following:

- **Maxwell’s divergence-free condition**: In a source-free region, the electric field ${\textbf{E}}$ must be divergence-free. For a beam propagating along the *y*-axis (${\textbf{k}} \parallel \hat{y}$), this constraint relates the longitudinal component ($E_y$) to the transverse components ($E_x, E_z$):
  14 $$\begin{aligned} \nabla \cdot {\textbf{E}} = \frac{\partial E_x}{\partial x} + \frac{\partial E_y}{\partial y} + \frac{\partial E_z}{\partial z} = 0 \end{aligned}$$
- **Longitudinal component generation (Paraxial approximation)**: In the paraxial approximation, where the longitudinal wave number $k_y \approx k_0$, the equation above is solved for $E_y$. The longitudinal field is thus proportional to the transverse divergence of the transverse field ${\textbf{E}}_\perp = (E_x, E_z)$:
  15 $$\begin{aligned} E_y \propto \frac{i}{k_y} \left( \frac{\partial E_x}{\partial x} + \frac{\partial E_z}{\partial z} \right) = \frac{i}{k_y} (\nabla _\perp \cdot {\textbf{E}}_\perp ) \end{aligned}$$
- **Topological charge reduction via gradient**: The input field $E_x$ carries the topological charge *M*, often represented by the azimuthal phase factor $f_M(x, z) = (x+iz)^M$. The required spatial differentiation (gradient) on this factor directly results in the $M \rightarrow M-1$ reduction:
  16 $$\begin{aligned} \frac{\partial E_x}{\partial x} \propto \frac{\partial }{\partial x} (x + i z)^M = M (x + i z)^{M-1} \end{aligned}$$
  This shows that the generated components ($E_y$, and similarly the crystal-induced $E_z$) scale with the reduced azimuthal order:
  17 $$\begin{aligned} E_{y/z} \propto (x + i z)^{M-1} \propto e^{i (M-1) \phi _{xz}} \end{aligned}$$

### Experimental feasibility and verification

The results presented in this work provide a systematic mapping of how both the propagation angle $\theta$ and the topological charge *M* jointly determine the evolution of all electric-field components inside a uniaxial crystal. While earlier studies have primarily examined either on-axis or strictly orthogonal propagation, the continuous angular dependence explored here has not been comprehensively investigated. Nevertheless, several features predicted by our model–such as field-component modulation, longitudinal–transverse coupling, and vortex-order transformation–are directly accessible with existing experimental techniques in structured-light and anisotropic-crystal optics. Below, we outline a feasible configuration for verifying the reported effects.

(i) **Beam generation and input conditions:** Vortex-Gaussian beams with tunable topological charge *M* can be generated using phase-only spatial light modulators (SLMs) or liquid-crystal q-plates. These devices provide full control over the vortex order and allow precise preparation of linearly polarized input beams. The beam waist, wavelength, and polarization can be matched to the simulation parameters to ensure direct comparability.
(ii) **Crystal orientation and angular control:** A rutile crystal of thickness *L* may be mounted on a precision rotation stage enabling continuous adjustment of the propagation angle $\theta$ with respect to the optical axis. This setup allows the experimental exploration of the full angular range considered in our analysis. The inclination can be monitored with sub-degree accuracy to maintain reproducibility and to probe the angular trends described in the theoretical results.
(ii) **Measurement of field components:** The transverse components $E_x$ and $E_z$ at the crystal exit face can be measured using polarization-resolved imaging techniques. Full-Stokes polarimetry or digital holography may be employed to reconstruct both amplitude and phase distributions. The longitudinal component $E_y$–though not directly measurable–can be retrieved using well-established vector-field reconstruction methods based on interferometric or polarization-diversity measurements. Thus, the predicted intensity maps, phase distributions, and Stokes-parameter patterns are all experimentally accessible.
(iii) **Connection to previous experimental studies.** The experimental observability of field-component modulation and polarization transformation in uniaxial crystals is supported by earlier works, including the spin–orbit conversion measurements of Brasselet *et al.*^7^, the analytical and experimental study of Gaussian-beam propagation by Shvedov *et al.*^8^, and the oblique-incidence investigations of Karpinski *et al.*^9^. These studies provide experimentally verified baselines on which the present work builds.
(iv) **Relevance and experimental impact:** The joint $(M,\theta )$ dependence identified in this work offers experimentally testable predictions regarding the amplification of longitudinal fields, the angular modulation of induced components, and the robustness of the $(M-1)$ spiral phase structure. These features are directly relevant to crystal-based vector-beam shaping, spin–orbit and orbital–orbital coupling mechanisms, and structured-light control in birefringent media. The framework developed here therefore provides not only theoretical insight but also clear guidance for future experiments involving vortex beams at arbitrary propagation angles in uniaxial crystals.

## Conclusions

This work presents significant insights into the complex interactions between structured light and anisotropic media. Our key findings demonstrate that propagation within the crystal substantially modifies the input beam. We observed significant beam shape distortion, with ellipticity being most pronounced at $\theta =0^\circ$. For the studied 1000 $\mu$m rutile crystal, the beam expands considerably, with an expansion factor of approximately 3.35 that is notably independent of the initial topological charge (*M*). Crucially, substantial longitudinal ($E_y$) and transverse ($E_z$) electric field components are generated from the initially purely *x*-polarized input beam. The relative intensities $S^r_y$ and $S^r_z$ of these generated components increase markedly with higher topological charge *M*. Their peak intensities also exhibit strong dependence on the propagation angle $\theta$, with $S^r_y$ maximizing close to $\theta =90^\circ$ and $S^r_z$ maximizing nearby $\theta =0^\circ$, showing complex oscillatory behavior at intermediate angles. Furthermore, the topological charge *M* distinctly imprints itself onto the phase structure: the primary *x*-component phase ($\phi _x$) retains the characteristic *M* intertwined spirals, while the generated components’ phases ($\phi _y, \phi _z$) exhibit $M-1$ spirals (for $M>1$). This study reveals the complex interplay between the input beam’s structure (topological charge *M*) and its propagation direction ($\theta$) relative to the optical axis in uniaxial crystals. Both parameters critically influence the resulting optical field distribution, including the generation and behavior of non-input polarization components, which must be carefully considered for advanced optical applications in anisotropic media. The generation of a significant, M-dependent longitudinal field component ($E_y$) is highly relevant for optimizing optical trapping and particle manipulation techniques. The pronounced angular dependence ($\theta$) of the generated components and the overall beam profile is critical for applications requiring precise phase-matching, such as second-harmonic generation or other nonlinear processes within anisotropic crystals. Moreover, the robust M-dependent phase structures could be exploited for encoding information in optical communication systems or for tailoring light fields used in the preparation and manipulation of quantum states.

## Data Availability

All data generated or analysed during this study are included in this published article.
